# Postura craneocervical de rocabado-penning en pacientes de ortodoncia

**DOI:** 10.21142/2523-2754-1203-2024-208

**Published:** 2024-09-17

**Authors:** Sofía Ramírez, Clarisse Díaz-Reissner, Clara Maldonado, Elena Jolay, Marta Ferreira-Gaona, Ana Fatecha

**Affiliations:** 1 Instituto Latinoamericano de Estudios Superiores. Asunción, Paraguay.sofiacano83@gmail.com , cdiazr@founa.edu.py , draclaritamr@gmail.com , elena.jolay@gmail.com Instituto Latinoamericano de Estudios Superiores Asunción Paraguay sofiacano83@gmail.com cdiazr@founa.edu.py draclaritamr@gmail.com elena.jolay@gmail.com; 2 Facultad de Odontología, Universidad Nacional de Concepción. Concepción, Paraguay.martaf.baez@gmail.com, anafatecha@founa.edu.py Universidad Nacional de Concepción Facultad de Odontología Universidad Nacional de Concepción Concepción Paraguay martaf.baez@gmail.com anafatecha@founa.edu.py

**Keywords:** bipedestación, maloclusión, radiografía dental, standing, malocclusion, radiography, dental

## Abstract

**Introducción:**

El equilibrio en bipedestación del cráneo sobre la columna en su porción cervical es fundamental para el diagnóstico de trastornos y alteraciones craneomandibulares en pacientes de todas las edades. No obstante, no había sido tomado en cuenta en el protocolo de diagnóstico diferencial, pese a que las alteraciones y disfunciones biomecánicas tanto en tejidos duros como blandos se evidencian en una telerradiografía lateral de cráneo.

**Objetivo:**

Describir la postura craneocervical con el análisis de Rocabado-Penning, en pacientes que acudieron a consulta en el Instituto Latinoamericano de Estudios Superiores (ILES).

**Metodología:**

El estudio fue observacional descriptivo de corte transversal. El estudio anatomo-radiológico se realizó en pacientes seleccionados entre los años 2012 y 2019 que acudieron a un instituto privado para realizarse su tratamiento de ortodoncia. Fueron incluidas telerradiografías laterales de cráneo, en las que se observaron las 7 vértebras cervicales.

**Resultados:**

La muestra quedó conformada por 30 pacientes. El 66,7% fue de sexo femenino, el 96,7% fue del tipo braquifacial, el 63,4% fue de clase I esqueletal de Ricketts, el 63,4% presentó mordidas profundas, la posición del hueso hioides fue baja para el 63,4% y la profundidad de la columna cervical es rectificada en un 96,7%. No se encontró relación estadísticamente significativa entre las clases esqueletales y las distancias suboccipitales.

**Conclusión:**

En los pacientes de la muestra prevaleció la clase I, braquifacial, mordida profunda, hueso hioides bajo y columna cervical rectificada; esta última podría tener como consecuencia dolor, tinnitus u otras sintomatologías de no ser tratada a tiempo.

## Abstract (en)

**Introduction:**

The standing balance of the skull on the spine in its cervical portion is a factor of consideration for the diagnosis of cranio-mandibular disorders and alterations in patients of all ages without distinction, that is, it is essential to conceive an accurate diagnosis in children and adults. However, over time, this element had not been taken into account in the differential diagnosis protocol, despite the fact that biomechanical alterations and dysfunctions in both hard and soft tissues are evidenced in a lateral skull teleradiography, essentially requested to initiate treatment.

**Objective:**

To describe the cranio-cervical posture with the Rocabado-Penning analysis, in patients who come for consultation at the Instituto Latinoamericano de Estudios Superiores (ILES).

**Methodology:**

The study was a cross-sectional descriptive observational study. The anatomical-radiological study was carried out on selected patients from 2012 to 2019 who attended a private institute to undergo orthodontic treatment. Lateral skull radiographs were included where the 7 cervical vertebrae were observed.

**Results:**

The sample was made up of 30 patients. 66.7% were female, 96.7% were brachyfacial type, 63.4% had Ricketts skeletal Class I, 63.4% had deep bites, the position of the hyoid bone was low for 63. .4% and the depth of the cervical spine is rectified by 96.7%. No statistically significant relationship was found between skeletal classes and suboccipital distances.

**Conclusion:**

In the patients in the sample, class I prevailed, brachyfacial, deep bite, low hyoid bone and rectified cervical spine, the latter could result in pain, tinnitus or other symptoms if not treated in time.

## INTRODUCCIÓN

Las maloclusiones tienen una prevalencia de incluso el 88% dentro de la población, lo que las convierte en un problema de la salud pública, pues ocupan el segundo lugar en la lista de patologías bucales, después de la caries dental, según la Organización Mundial de la Salud OMS ^1^. No obstante, la postura craneocervical ha sido omitida en los protocolos de diagnóstico por varios años, aun cuando esta es la responsable de la estabilidad ortostática y puede constituir un factor influyente en las disfunciones del sistema estomatognático ^2^.

Dada la posición recta del cuerpo, la columna vertebral en humanos presenta una doble curvatura en forma de S, como característica principal. Las vértebras cervicales se ubican entre la base de cráneo y las vértebras dorsales. Se las divide en columna cervical alta (C1 y C2, cóndilos occipitales y foramen magno) y baja (C3 a C7). Con base en esto, Rocabado (1984) propone una técnica que permite la determinación de la estabilidad del cráneo en sentido anteroposterior y vertical, a través del análisis cefalométrico craneocervical en telerradiografías. Previamente, ya había establecido la asociación entre la maloclusión clase II y la postura adelantada de la cabeza ^3^.

El desequilibrio postural en esta clase esquelética puede deberse a el aumento de la actividad de la musculatura prevertebral, lo que verticaliza la columna cervical, al aumentar la tensión de los músculos infra y suprahioideos, lo que ocasiona un desplazamiento craneal en hiperextensión dorsal, manifestándose de esta manera las características sagitales, verticales y transversales de esta clase ^2^.

Si la ubicación del hueso hioides no es la adecuada, la musculatura no accionará sinérgicamente y, por lo tanto, en el sistema óseo no será visible una correcta relación entre la posición de la cabeza y la disposición morfológica craneofacial. Así, una posición anterior de la cabeza se acompaña de alteraciones funcionales que causan elevación y retrusión mandibular, lo cual posiciona la lengua hacia adelante. Por tanto, una mala posición de la cabeza altera relaciones biomecánicas craneocervicales y craneomandibulares, lo que contribuye a la mala postura corporal del individuo e influye negativamente en el crecimiento normal ^4^.

En presencia de flexión de la base craneal, se inhibe la elongación y se presenta un acortamiento de la base de cráneo, lo cual reduce o acorta su ángulo base de cráneo, y da lugar a una implantación anterior e inferior de la articulación, lo cual desplaza la mandíbula más adelante. Este proceso, en conjunto con factores genéticos y funcionales, puede desarrollar una maloclusión clase III ^5^.

Por lo expuesto, teniendo en cuenta que en los estudios efectuados en individuos con maloclusión clase II y clase III las variaciones morfológicas del patrón de crecimiento esqueletal están asociadas con el ángulo de la base del cráneo, y que la flexión o extensión de la base craneal podría influir en la posición del maxilar inferior, debido del desplazamiento de la cavidad glenoidea. Esto se debe a que, cuando se elonga la base del cráneo, se incrementa el ángulo de la base craneal, lo cual produce una implantación posterior y superior de la articulación temporomandibular que propicia el desplazamiento del maxilar inferior a una posición más posterior, y que, junto a otros factores, puede llevar a una maloclusión de clase II. Por esto, resulta importante realizar evaluaciones de posturología en nuestra población, considerando que no han sido publicados artículos similares sobre paraguayos.

Se planteó como objetivo de este estudio describir la postura craneocervical con el análisis de Rocabado-Penning en un grupo de pacientes paraguayos utilizando telerradiografías laterales de cráneo.

## MATERIALES Y MÉTODOS

El estudio fue observacional descriptivo de corte transversal. Participaron 30 pacientes (33,3% varones, 66,7% mujeres) que contaron con telerradiografías laterales de cráneo del Instituto Latinoamericano de Estudios Superiores (ILES), en Asunción (Paraguay), entre los años 2012 y 2019. Fueron incluidas telerradiografías laterales de cráneo donde se observaron las 7 vértebras cervicales.

Se midieron las variables patrón esquelético de Ricketts, postura craneocervical de Rocabado (ángulo cráneo cervical, distancia C0-C1, distancia C1-C2 y posición hioides) y postura cervical de Penning (profundidad de la columna cervical).

El patrón esquelético de Ricketts se consideró la convexidad facial (clase I: 1 mm-4 mm, clase II: > 4 mm, clase III: < 1 mm), el ángulo craneocervical (rotación posterior del cráneo: < 96°, norma: 96°-106°, rotación anterior del cráneo: > 106°), la distancia C0-C1 y la distancia C1-C2 (disminución del espacio suboccipital: < 4 mm, norma: 4-9 mm), la posición del hioides con respecto a la línea C3-RGn (alto, medio y bajo) y la posición de la curvatura cervical (cifótica: < 2 mm, rectificada: 2 mm-7 mm, normal: 8 mm-12 mm, lord < 12 mm) ^6^.

Para la interpretación espacial y posicional de la columna cervical se consideró lo siguiente: a) alto o negativo: el hueso hioides está por encima del plano C3 - RGn; b) Promedio: si pasa sobre o coincide con en el plano C3 - RGn; y c) Bajo: el hueso hioides está por debajo del plano C3 - RGn.

Se trazó una línea tangente que recorre el margen posterosuperior del proceso odontoides y va hacia el punto más posterior e inferior del cuerpo de la última vértebra cervical. Luego, se tomó como referencia de equilibrio la cuarta vértebra cervical y se traza una línea perpendicular a la tangente antes trazada, y se realiza la medición de esta línea. El promedio de profundidad normal es de 10 ± 2 mm, y se consideró lo siguiente: a) Rectificada, con valores menores a 8 mm; b) Cifótica, cuando los valores son cifras negativas; y c) Lordótica, con valores mayores a 12 mm.

El protocolo de investigación fue aprobado por el Comité de Ética en Investigación de la Universidad Nacional de Asunción, con el Informe N.º 27/22. Los datos de los pacientes fueron tratados de manera confidencial y solo utilizados para los fines del estudio.

Se aplicó estadística descriptiva e inferencial. Se utilizó la prueba chi-cuadrado de Pearson para relacionar la clase esqueletal con las distancias del primer y segundo espacio occipital (reagrupadas en Norma y Aumentado/Disminuido); pero no se pudo aplicar el test exacto de Fisher para relacionar la clase esqueletal (reagrupadas en clase I y clases II/III) con la profundidad de la columna cervical (rectificada y cifótica), debido a que el 50% de las casillas contienen valores esperados inferiores a 5. Se utilizó un nivel de confianza del 95%, mediante el programa estadístico IBM SPS Statistics versión 25.

## RESULTADOS

La muestra quedó conformada por 30 pacientes. El 66,7% fueron mujeres y el 33,3%, varones. De la muestra seleccionada, el tipo braquifacial representa el 96,7% y el mesofacial, el 3,3%. En cuanto al patrón esqueletal de Ricketts, en la muestra encontramos un 63,4% de clase I, un 33,3% de clase II y un 3,3% de clase III. Las mordidas profundas representan el 63,4% de la muestra, el 33,3% es abierta y el 3,3% es normal. La posición del hueso hioides es baja para el 63,4% del total de la muestra, promedio para el 3,3%, alto para el 16,7% y lineal para el 16,7%.

**Figura 1:**
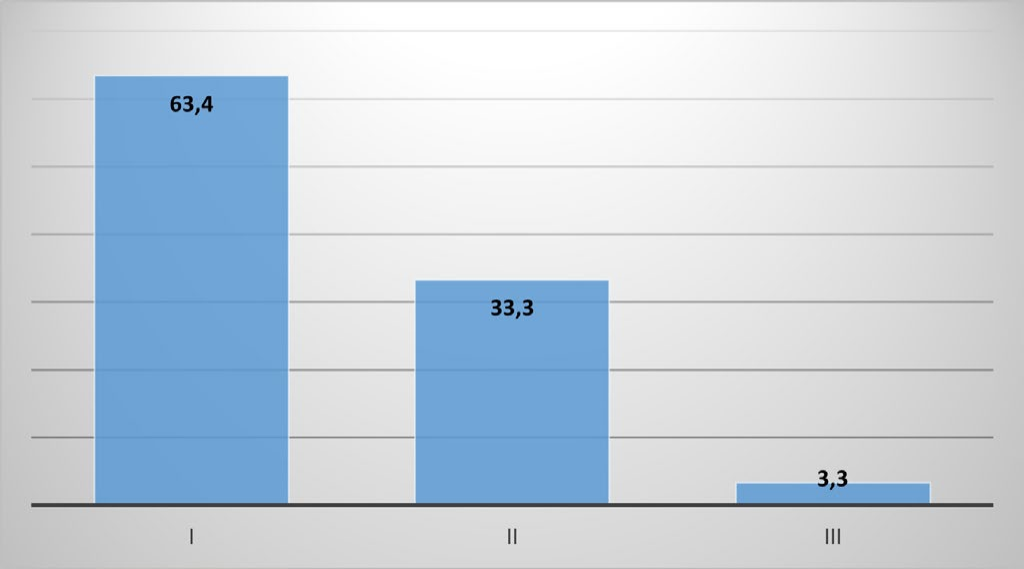
Clase esqueletal de Ricketts

Para la muestra, la profundidad de la columna cervical es rectificada en un 96,7% de los pacientes seleccionados y en el 3,3% es cifótica. La postura craneocervical está en la norma para el 63,3% de la muestra, es anterior para el 16,7% y posterior para el 20,0%.

**Figura 2:**
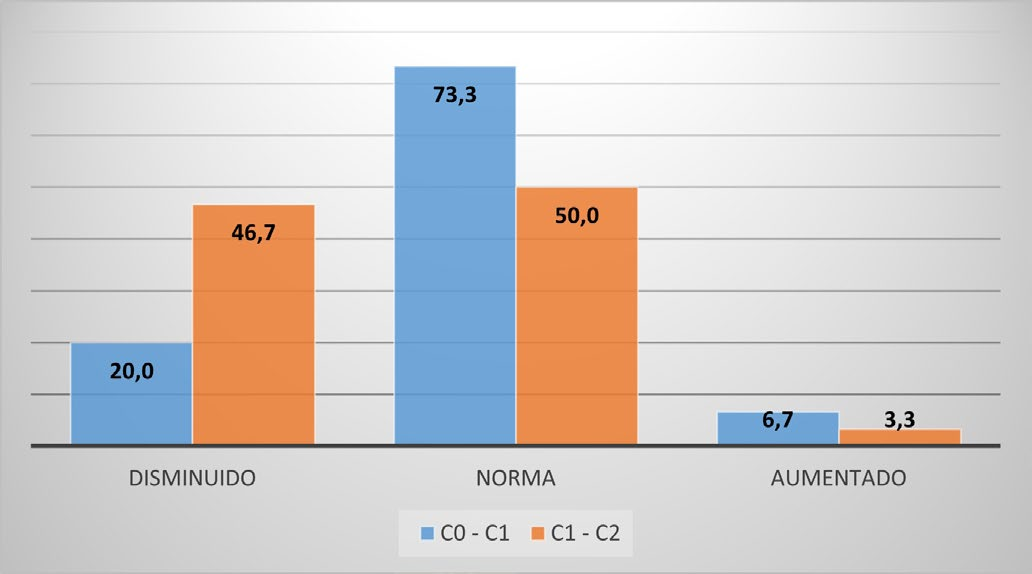
Distancias del primer espacio suboccipital (C0_C1) y el segundo espacio suboccipital (C1_C2)

Las distancias del primer espacio suboccipital y del segundo espacio suboccipital se encuentran en la norma (Figura 2).

No se encontró una relación estadísticamente significativa entre la distancia fuera de norma del primer espacio suboccipital y la clase esqueletal; más bien, se observa que la distancia del primer espacio se encontraba en la norma y en su mayoría eran de clase I (Tabla 1).

**Tabla 1:** Relación entre la clase esqueletal y la distancia del primer espacio suboccipital

Primer espacio suboccipital	Clase I	Clase II/III	Total
Norma	15 (50,0%)	7 (23,3%)	22 (73,3%)
Disminuido/aumentado	4 (13,3%)	4 (13,3%)	8 (26,7%)
Total	19 (63,3%)	11 (36,7%)	30 (100,0%)

X^2^ = 0,835; gl = 1; p = 0,361

La mitad de los pacientes presentaron el segundo espacio suboccipital fuera de la norma, pero más de la mitad eran clase I, por lo que no se encontró una relación estadísticamente significativa entre ambos (Tabla 2).

**Tabla 2:** Relación entre la clase esqueletal y distancia del segundo espacio suboccipital

Segundo espacio suboccipital	Clase I	Clase II/III	Total
Norma	8 (26,7%)	7 (23,3%)	15 (50,0%)
Disminuido/aumentado	11 (36,7%)	4 (13,3%)	15 (50,0%)
Total	19 (63,3%)	11 (36,7%)	30 (100,0%)

X^2^ = 1,292; gl = 1; p = 0,256

## DISCUSIÓN

Se realizó un estudio con radiografías con el objetivo de describir la postura craneocervical con el análisis de Rocabado-Penning en pacientes que acuden a consulta en el Instituto Latinoamericano de Estudios Superiores (ILES). Se encontró un 63,3% con clase I, estando el 36,7% en norma en relación con el ángulo craneocervical; el 40,0% con una posición baja del hueso hioides, estando en norma el 50,0% del primer espacio suboccipital y el 33,3% del segundo espacio. Se debe tener en cuenta que los pacientes fueron seleccionados por casos consecutivos considerando prioritariamente la presencia de la séptima vértebra cervical en la radiografía. Como el estudio es retrospectivo, muchas radiografías tuvieron que ser descartadas, ya que no es habitual que el ortodoncista solicite específicamente en la orden de estudios la presencia de dichas vertebras, así como tampoco los radiólogos acostumbran considerar ese recaudo. Por tanto, no pudieron ser tomados en cuenta el patrón esquelético de Ricketts como criterio de selección, ni la edad ni el sexo.

Hay que considerar que la maloclusión de clase II está asociada con alteraciones en la posición cervical, por lo que predomina la anteroversión con alteración en la columna vertebral, mayormente la escoliosis ^7^. En este estudio, solo el 33,3% fueron de clase II; por tanto, no fue posible evaluar dichas asociaciones estadísticas. Sin embargo, el 63,4% presentó una posición del hioides baja y el 96,7% tenía la columna rectificada, lo que describe una elevada presencia de alteraciones. Cada vez se hace más evidente la relación que guarda la oclusión con la postura corporal ^8^. 

No obstante, en un estudio realizado por Aldana *et al*. ^9^ en pacientes chilenos, se concluyó que la asociación entre la maloclusión y la postura craneocervical resultó estadísticamente débil. Aunque actualmente sigue siendo controversial, en una revisión bibliográfica se determinó que la mayoría de los estudios evaluados mostraron una relación entre la presencia de cambios oclusales y posturales, pero aún no es posible dilucidar cómo esto afecta el diagnóstico y el tratamiento ortodóntico ^10^. Estudios recientes sugieren que señales aferentes de la oclusión dental contribuyen eficazmente al control del equilibrio cuando hay perturbaciones externas, es decir, la superficie de apoyo inestable, la fatiga y las tareas que se realizan ^11^.

Por otro lado, se ha encontrado que trabajadores de ensamblaje de productos electrónicos tuvieron una alta prevalencia de trastornos musculoesqueléticos (TME), y que la flexión/extensión del cuello durante mucho tiempo fue una de las variables relacionadas con dichos TME ^12^. Asimismo, tatuadores experimentaron malestar en el cuello que empeoraba con las actividades laborales ^13^ y en el caso de cirujanos la prevalencia de trastornos de cuello fue del 6% al 74% ^14^. Finalmente, en una revisión sistemática, se concluyó que existe una correlación positiva entre la flexión del cuello y los trastornos del cuello en trabajadores ^15^. En cuanto a la presencia de dolor, la prevalencia de este en el cuello, relacionado con el trabajo entre los operadores de máquinas de coser, fue del 45,8% ^16^, mientras que la tasa de prevalencia del dolor de cuello fue significativamente mayor en odontólogos (73%) en comparación con los controles (52%) ^17^. Con esto se pretende demostrar que la postura y el dolor cervical pueden verse afectados por el tipo de actividad laboral en adultos.

El uso de teléfonos inteligentes aumentó los ángulos de flexión de la cabeza y el cuello en todas las posturas, y sentarse sin apoyo para la espalda mostró mayores ángulos de flexión de cabeza y el cuello ^18^. El uso de dispositivos informáticos, especialmente teléfonos móviles, y el aumento de la flexión de la columna cervical indican que los problemas vertebrales cervicales aumentarán incluso en las personas más jóvenes en el futuro ^19^. También en niños y adultos ya se pueden observar alteraciones; por tanto, resulta importante para el ortodoncista tener en cuenta la observación de las vértebras cervicales en sus pacientes. 

En este estudio, la presencia de la columna cervical rectificada fue del 96,7% y la cifótica, del 3,3%; mientras que en niños y adultos israelíes el 51% era lordótico; el 41%, rectificada; y el 10%, cifótica ^20^. Llama la atención la elevada cifra obtenida en este estudio, pero se debe tener en cuenta que la población objeto del análisis es reducida, por lo que esto podría variar en estudios poblacionales.

### Limitaciones

Una limitación importante fue la cantidad de telerradiografías laterales de cráneo descartadas por no cumplir con el requisito principal de observar las 7 vértebras cervicales necesarias para realizar la medición de la columna cervical.

Tomando en cuenta lo mencionado, se recomienda a los profesionales que al solicitar la orden de estudios radiográficos hagan hincapié no solo en la posición natural de cabeza, sino también en la importancia de observar las 7 vértebras cervicales en las telerradiografías laterales de cráneo, para de esta manera evaluar la estabilidad de la cabeza sobre la columna cervical.

## CONCLUSIÓN

En los pacientes de la muestra prevaleció la clase I, braquifacial, mordida profunda, hueso hioides bajo y columna cervical rectificada; esta última podría tener como consecuencia dolor, tinnitus u otras sintomatologías de no ser tratada a tiempo.

## Fuente de financiamiento:

Autofinanciado
